# Supplementary material for: Breast cancer incidence and predictions (Monastir, Tunisia: 2002–2030): A registry-based study
Source: PLoS One. 2022 May 26;17(5):e0268035. doi: 10.1371/journal.pone.0268035 (PMC9135193; doi:10.1371/journal.pone.0268035)
Supplement: Attachment — Submitted filename: Response-to-Reviewers.docx [file pone.0268035.s002.docx]

**Response to Reviewers**

Dear Editor,

Thank you for your attention to our work. The comments and suggestions we have received are valuable and very helpful to improve our manuscript. We have made revisions based on your latest comments and suggestions, as described in the authors' response.

We respond in detail to each of the reviewers’ comments. They raised important issues and we agree with almost all their comments. We have revised our manuscript according to their indications. We hope that they will find our responses to their comments satisfactory, and we are willing to finish the revised version of the manuscript including any further suggestion that the reviewers may have. The revision has been developed in consultation with all coauthors, and each author has given approval to the final form of this revision. All changes made in the revised version will be visible in red.

Please find as attached files:

The “Response to Reviewers”,

the “'Revised Manuscript with Track Change” and

the “Manuscript” (the unmarked version of the revised manuscript).

We sincerely hope that the revision of the manuscript will be satisfactory and the enclosed version will be acceptable for publication.

Once again thank you for your cooperation.

Sincerely Yours,

Dr Imen ZEMNI

**Journal Requirements:**

1. Please ensure that your manuscript meets PLOS ONE's style requirements, including those for file naming.

**Response:** Thank you. We did the required modifications.

2. In your Data Availability statement, you have not specified where the minimal data set underlying the results described in your manuscript can be found.

**Response:** Thank you. We attached an Excel supplementary file intitled “S1 File. Female breast cancer new cases in Monastir between 2002 and 2013” in Supporting Information. (Lines 461 and 462)

3. PLOS requires an ORCID iD for the corresponding author in Editorial Manager on papers submitted after December 6th, 2016. Please ensure that you have an ORCID iD and that it is validated in Editorial Manager.

**Response:** Thank you. We indicated the ORCID iD of the corresponding author.

4. Please review your reference list to ensure that it is complete and correct.

**Response:** Thank you. We reviewed the reference list and added other references as it was recommended by reviewer 2.

**Review Comments:**

**Reviewer 1:**

1. Abstract: The second line "Among females'.....causing mortality" needs rephrasing.

**Response:** Thank you. We did the necessary modifications. (Lines 32 and 33)

2. For the worldwide readers, it would be better that the spelling of "Crud" as in crude incidence rate be written uniformly as "Crude".

**Response:** Thank you. We did the necessary modifications. (Lines 82,113,141,142,145,149 and 305)

3. In methodology section, As you have included only females with new breast cancer in your study, (Male breast cancer cases were excluded), it is not clear that you used sex specific population as denominator or not. The title of the relevant tables should also mention the word "females".

**Response:** Thank you. As we have included only females with new breast cancer in our study, we used female population as denominator. We added this information in the methodology section (Lines 115 and 116). We also mentioned the word "female" in the titles of the relevant tables (Lines 155,161 and 179).

4. The time point of the population used as denominator (eg mid-year...or something else ) is not mentioned.

**Response:** Thank you. We computed the point incidence rate of breast cancer (per 100,000 population) for each year based on the Tunisian National Institute of Statistics data. Incidence rates were expressed per 100,000 person-years: The Tunisian National Institute of Statistics carries out censuses of the Tunisian population every 10 years. The last censuses were carried out between April and May 2014. Data on the population estimate in other years are given through projections made by the Tunisian National Institute of Statistics.

5. Statistical analysis section should mention the full details of the SPSS software used like the city, country etc.

**Response:** Thank you. We did the necessary modifications. (Lines 112 and 113)

6. In discussion section, Spelling of "contralateral"

**Response:** Thank you. We corrected the spelling. (Line 276)

**Reviewer 2:**

1. The methodology is adequate, but I could not locate the ethics committee recommendations or consent.

**Response:** Thank you. We mentioned in the methodology section that the protocol of this study was approved by the ethics Committee of the faculty of medicine of Monastir (Reference Number: IORG0009738N°101/OMB0990-0279).) (Lines 124,125 and 126)

2. The discussion in this paper is a momentous tasks and needs more trends to be discussed worldwide, although the authors have tried to reason well that why Monastir has higher breast cancer incidence rate in comparison to the national rate and how does it stand globally. May I suggest more scientific discussions to the authors. You are also requested to compare with predictions from Machine Learning Models.

**Response:** Thank you. We revised the discussion and we added paragraphs to discuss:

- The incidence rate in Monastir comparatively to other national results and international findings (lines 201-211).
- Trends of breast cancer incidence comparatively to last reports from Tunisia and to statistics from the different regions in the World (Lines 230-257).
- Trends according age groups (Line 258-267).

3. There were no limitations and biases mentioned in the paper and authors are requested to add them as well.

**Response:** Thank you. We added a paragraph for limitations at the end of the manuscript (Lines 279-287).

4. Spelling errors noted (crud = crude)

**Response:** Thank you. We did the necessary modifications. (Lines 82,112,136,137,144 and 264)
